# Title, Table of Contents and Acknowledgements

**DOI:** 10.1080/26410397.2021.2145080

**Published:** 2022-12-07


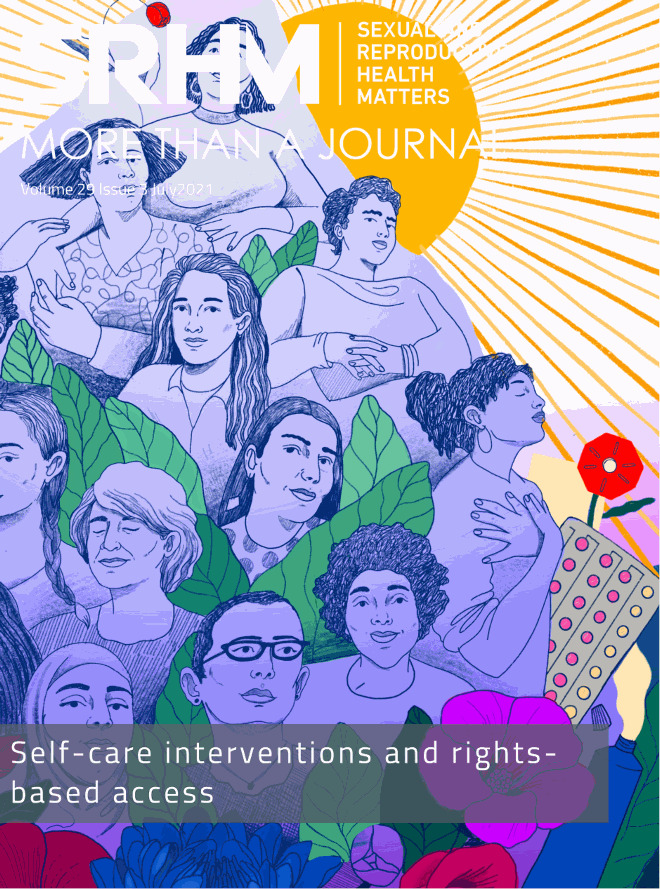


**Editorial**

1 Laura Ferguson *and* Manjulaa Narasimhan   Centring rights-based access to self-care interventions

**Commentaries**

8 Caitlin Corneliess*,* Katelin Gray*,* Jennifer Kidwell Drake*,* Allen Namagembe*, *Aurora Stout *and* Jane Cover   Education as an enabler, not a requirement: ensuring access to self-care options for all

14 Denis Kibira, Victoria Boydell, Lillian Mworeko, James Kiarie   Considerations for social accountability in the expansion of self-care for sexual and reproductive health and rights

20 Génesis Luigi-Bravo, Kaur Roopan Gill   Safe abortion within the Venezuelan complex humanitarian emergency: understanding context as key to identifying the potential for digital self-care tools in expanding access

28 Juliette Ortiz, Sandra Salazar, Tatiana Lesmes, Eliana Marulanda, Maria Mercedes Vivas   Regulatory authorities are limiting telemedicine’s potential to deliver legal abortion care to everyone in Colombia

**Reviews**

32 Holly M. Burke, Kathleen Ridgeway, Kate Murray, Alexandria Mickler, Reana Thomas, Katie Williams   Reproductive empowerment and contraceptive self-care: a systematic review

82 Caitlin E. Kennedy, Ping Teresa Yeh, Jack Byrne, L Leigh Ann van der Merwe, Laura Ferguson, Tonia Poteat, Manjulaa Narasimhan   Self-administration of gender-affirming hormones: a systematic review of effectiveness, cost, and values and preferences of end-users and health workers

99 Caitlin E. Kennedy, Ping Teresa Yeh Jingjia Li, Lianne Gonsalves, Manjulaa Narasimhan   Lubricants for the promotion of sexual health and well-being: a systematic review

**Research articles**

121 Chiara Bercu, Heidi Moseson, Julia McReynolds-Pérez, Emily Wilkinson Salamea, Belén Grosso, María Trpin, Ruth Zurbriggen, Carolina Cisternas, Milena Meza, Viviana Díaz, Katrina Kimport   In-person later abortion accompaniment: a feminist collective-facilitated self-care practice in Latin America

144 Laura Ferguson, Manjulaa Narasimhan, Jose Guitierrez, William Jardell, Sofia Gruskin   Law, human rights and gender in practice: an analysis of lessons from implementation of self-care interventions for sexual and reproductive health

166 Camille Garnsey, Alexandra Wollum, Sofía Garduño Huerta, Oriana López Uribe, Brianna Keefe-Oates, Sarah E Baum   Factors influencing abortion decisions, delays, and experiences with abortion accompaniment in Mexico among women living outside Mexico City: results from a cross-sectional study

181 Rebecca Hémono, Laura Packel, Emmyson Gatare, Laura Baringer, Nicole Ippoliti, Sandra I. McCoy, Rebecca Hope   Digital self-care for improved access to family planning and reproductive health services among adolescents in Rwanda: preliminary findings from a pilot study of CyberRwanda

198 Amanda Kalamar, Christine Bixiones, Grace Jaworski, Klaira Lerma, Melvin Mwansa, Rachel Lawreh, Selase Adjei   Supporting contraceptive choice in self-care: qualitative exploration of beliefs and attitudes towards emergency contraceptive pills and on-demand use in Accra, Ghana and Lusaka, Zambia

213 Sara Larrea, Camila Hidalgo, Constanza Jacques- Aviñó, Carme Borrell, Laia Palència   “*No one should be alone in living this process*”: trajectories, experiences and user’s perceptions about quality of abortion care in a telehealth service in Chile

226 Carmen Logie, Isha Berry, Laura Ferguson, Kalonde Malama, Holly Donkers, Manjulaa Narasimhan   Uptake and provision of self-care interventions for sexual and reproductive health: findings from a global values and preferences survey

247 Francis Obare, Fatou Mbow, Saumya RamaRao, Avishek Hazra   Husbands' concerns and experiences of the progesterone vaginal ring in three sub-Saharan African countries: a mixed methods study

265 Swastika Shrestha, Saki Thapa, Paul Sims, Andreea Ardelean, Anamika Basu, Maxine Caws, Suman Chandra Gurung, Gillian Holdsworth   Feasibility of an HPV self-sampling pathway in Kathmandu Valley, Nepal using a human-centred design approach

280   Correction

**Executive Editor:** Emma Pitchforth

**Chief Executive**: Eszter Kismödi

**Senior Editors:** Sarah Keogh, TK Sundari Ravindran

**Managing Editor**: Pete Chapman

**Monitoring Editor**: Pathika Martin

**South Asia Hub Manager**: Sanjeeta Gawri

**Communications Manager**: Alexane Bremshey

**Operations Manager**: Amy Griffiths

**Associate Editors:** Laura Ferguson, Atsumi Hirose, Nambusi Kyegombe, Helen Potts, Mindy Jane Roseman, Nina Sun, Joyce Wamoyi

**Editorial committee for this issue:** Mauro Cabral, Georgina Caswell, Laura Ferguson, Eszter Kismödi, Priya Nanda, Manjulaa Narasimhan

**Funding:** We thank the Children’s Investment Fund Foundation for supporting the publication of this issue.

**Cover image:** *Untitled* (Digital print). © Rose Jaffe

**Peer reviewers:** Antonia Biggs, Kelly Blanchard, Rebecca Callahan, Lidia Cecilia Casas, Venkatesan Chakrapani, Sruthi Chandrasekaran, Megan Christofield, Jane Cover, Catherine Dodds, Ilana Dzuba, Sandra Fernández, Tamara Fetters, Katharine Footman, Evelyn Fuentes-Rivers, Katherine Gambir, Roopan Gill, Celia Karp, Tamil Kendall, Leah Kenny, Amy Krauss, Stephanie Küng, Rachel Lenzi-Weisbecker, Shelly Makleff, Julia McReynolds-Perez, Pierre Moon, Simon Mwima, Priya Nanda, Jitendra Pariyar, Anne Philpott, Lucia Berro Pizzarossa, Bil Powell, Saumya RamaRao, Eusebio Rubio, Cianán Russell, Dana Sarnak, Marta Schaaf, Patty Skuster, Aamod Dhoj Shrestha, Tonny Ssekamatte, Bianca Maria Stifani, Joe Strong, Verónica Undurraga, Heather Vahdat, Rachel Lenzi-Weisbecker, T Charles Witzel, Shannon D Wood, Phyu Phyu Thin Zaw

**Translation:** Françoise de Luca-Lacoste translated abstracts from English to French and Lisette Silva translated abstracts from English to Spanish.

**Copyright ©2022 Sexual and Reproductive Health Matters:** This is an Open Access journal distributed under the terms of the Creative Commons Attribution License (http://creativecommons.org/licenses/ by/4.0/), which allows for sharing and adapting the work for any purpose, even commercially, provided appropriate credit is given with a link to the originally published item, a reference to the author(s) and links to their homepages, reference to the license under which the article is published and a link to this, as well as an indication of any changes that have been made to the original. ISSN (Online) 2641-0397

www.srhm.org / www.srhmjournal.orgTwitter @SRHMJournalFacebook @SRHMJournal

